# Associations of Serum Concentrations of Persistent Organic Pollutants with the Prevalence of Periodontal Disease and Subpopulations of White Blood Cells

**DOI:** 10.1289/ehp.11425

**Published:** 2008-07-03

**Authors:** Duk-Hee Lee, David R. Jacobs, Thomas Kocher

**Affiliations:** 1 Department of Preventive Medicine and Health Promotion Research Center, School of Medicine, Kyungpook National University, Daegu, Korea; 2 Division of Epidemiology, School of Public Health, University of Minnesota, Minneapolis, Minnesota, USA; 3 Department of Nutrition, University of Oslo, Oslo, Norway; 4 Unit of Periodontology, School of Dentistry, Ernst Moritz Arndt University Greifswald, Greifswald, Germany

**Keywords:** immune system, neutrophil, organochlorine pesticides, periodontitis, persistent organic pollutants

## Abstract

**Background:**

Persistent organic pollutants (POPs), which are endocrine disruptors that accumulate in adipose tissue, can increase the risk of periodontal disease through the disturbance of the immune system.

**Objective:**

We examined associations of background exposure to POPs with periodontal disease in the general population.

**Design:**

Cross-sectional associations of concentrations of serum POPs with the prevalence of periodontal disease were investigated in 1,234 adults ≥ 20 years of age in the National Health and Nutrition Examination Survey 1999–2002.

**Results:**

Among several POPs, organochlorine (OC) pesticides were most strongly associated with periodontal disease. Adjusted odds ratios across quintiles of OC pesticides were 1.0, 1.3, 1.7, 2.4, and 2.7 (*p* for trend < 0.01) for the presence in any site of clinical attachment loss ≥ 4 mm and 1.0, 1.7, 2.6, 3.4, and 3.7 (*p* for trend < 0.01) for the presence of pocket depth ≥ 4 mm. Polychlorinated biphenyls and polychlorinated dibenzo-*p*-dioxins also showed significant positive associations with one or both definitions of periodontal disease. Results did not materially change when continuous variables of clinical attachment loss or pocket depth were used as outcomes. Although participants with periodontal disease had higher white blood cell and neutrophil counts, neutrophil counts were inversely related to OC pesticides (*p* for trend < 0.01). These inverse associations did not change after excluding subjects with C-reactive protein ≥ 3 mg/L.

**Conclusion:**

POPs, especially OC pesticides, were positively associated with periodontal disease, possibly through immunomodulation due to OC pesticides.

Periodontitis, a common disease caused by oral bacterial infection and inflammation, leads to irreversible attachment loss, bone destruction, and eventually tooth loss. The current widely accepted paradigm about pathogenesis of periodontitis is that the destruction observed in periodontitis is the result of an improperly regulated immune response to bacterial infection rather than the directly destructive effect of the bacterial pathogens themselves (Van Dyke and Serhan 2003).

Two recent studies have reported that a polymorphism of *N*-acetyltransferase 2, an enzyme involved in phase II metabolism of xenobiotics, is associated with periodontitis (Kocher et al. 2002; Meisel et al. 2002); subjects with the slow-acetylating genotype had more severe periodontal disease. These studies were designed to examine whether varying *N*-acetyltransferase enzyme activity according to *N*-acetyltransferase polymorphism differentially affected the periodontal risk due to cigarette smoking through the consequent effect on metabolism of smoke-derived xenobiotics. Although interpretation of these studies mainly stressed the importance of genetic susceptibility in the pathogenesis of periodontitis, the findings apply generally to a role of any xenobiotic in the pathogenesis of periodontitis. Supporting this idea, recent studies have reported that blood lead concentrations associated positively with periodontal disease (Dye et al. 2002; Saraiva et al. 2007). Although in one study the association was interpreted as lead released after bone destruction due to periodontal disease (Dye et al. 2002), lead could be a causal agent for periodontal disease. Lead is a xenobiotic that can increase the risk of periodontal disease, and exposure to a heavy metal such as lead affects the immune system (Dietert and Piepenbrink 2006). In addition, workers exposed to acid fumes or chemicals in an oil company reported a higher risk of periodontal disease (Amin et al. 2001; Ruppe and Werckmeister 1989).

Possible candidates among xenobiotics that might cause periodontal disease are persistent organic pollutants (POPs)—endocrine disruptors that are highly toxic, persist in the environment, and bioaccumulate in fatty tissues of living organisms (Abelsohn et al. 2002). The general population without occupational exposure is exposed to POPs through food consumption (Abelsohn et al. 2002). POPs are primarily known to be immunosuppressive, although they can also increase autoimmune reactions (Ahmed 2000; Baccarelli et al. 2002; Banerjee 1999). Studies on specific clinical correlates have suggested increased prevalence of middle ear or other bacterial infections among exposed persons, especially children (Tsuji 2000; Weisglas-Kuperus et al. 2000). Thus, exposure to POPs may increase susceptibility to bacterial infection in the periodontium through the impairment of the immune system.

A series of recent epidemiologic studies has reported dose–response relationships between the background exposure to POPs and various clinical outcomes including type 2 diabetes, metabolic syndrome, or cardiovascular diseases (Ha et al. 2007; Lee et al. 2006, 2007a, 2007b), which are associated with periodontal disease (Demmer and Desvarieux 2006; Loe 1994). In particular, obesity was not associated with type 2 diabetes among subjects with very low concentrations of POP (Lee et al. 2006).

This study was performed with the hypothesis that there is an association between serum concentrations of POPs and the prevalence of periodontal disease. We also investigated the relationship of serum concentrations of POPs with subpopulations of white blood cells (WBC), with the hypothesis that background exposure to POPs was associated with an immunosuppressive effect.

## Materials and Methods

The 1999–2002 National Health and Nutrition Examination Surveys (NHANES) conducted by the Centers for Disease Control and Prevention (CDC) were designed to be nationally representative of the noninstitutionalized U.S. civilian population on the basis of a complex, multistage probability sample. Details of the NHANES protocol and all testing procedures are available elsewhere [National Center for Health Statistics (NCHS) 2006a, 2006b]. The study protocol was reviewed and approved by the CDC institutional review board; additionally, informed written consent was obtained from all subjects before they took part in the study. Serum concentrations of various biologically important POPs or their metabolites were measured in subsamples of the NHANES 1999–2002 surveys (NCHS 2005).

The NHANES standardized home interview was followed by a detailed physical examination. Venous blood samples were collected and shipped weekly at −20°C. POPs were measured by high-resolution gas chromatography/high-resolution mass spectrometry using isotope dilution for quantification. All these analytes were measured in approximately 5 mL serum. The POPs were reported on a lipid-adjusted basis using concentrations of serum total cholesterol and triglycerides.

Detailed information about the oral health component protocol have been described elsewhere (NCHS 2001). Briefly, the periodontal examination was conducted at two sites, midbuccal and mesiobuccal, for each tooth, in two randomly chosen quadrants, one maxillary and one mandibular, on the assumption that conditions in these two quadrants would represent the mouth. Third molars were excluded, so a maximum of 14 teeth and 28 sites per individual were examined. We used various definitions of periodontitis to see if there were consistent patterns of associations regardless of definition. Clinical attachment loss (CAL), an indicator of cumulative periodontal destruction, was defined at each site that had experienced a minimum of 4 mm of measured loss. Periodontal pocket depth (PD), an indicator of presence of active disease, was expressed in parallel. We also defined the percentage of sites affected by CAL ≥ 4 mm or a PD ≥ 4 mm, each with denominator the number of dental sites evaluated (CAL%4 or PD%4, respectively). The periodontal disease indicators were used as both continuous (CAL%4 and PD%4) and dichotomous variables [presence in any site vs. absence in all sites of CAL ≥ 4 mm (anyCAL4) or PD ≥4 mm (anyPD4)]. Use of other definitions of periodontitis (Demmer et al. 2008; Page and Eke 2007) did not change our results.

Although 49 POPs were measured in both NHANES 1999–2000 and 2001–2002, to avoid bias in prevalence odds estimation among those below the limit of detection (LOD), we selected the 19 POPs for which at least 60% of participants had concentrations > LOD: three polychlorinated dibenzo-*p*-dioxins (PCDDs), three polychlorinated dibenzofurans (PCDFs), four dioxin-like polychlorinated biphenyls (PCBs), five non–dioxin-like PCBs, and four organochlorine (OC) pesticides. There were 1,243 study participants ≥ 20 years of age with information on oral examination and serum concentrations of the 19 selected POPs.

For each POP, subjects with serum concentrations < LOD were the reference group, and subjects with detectable values were categorized by cutoff points of 25th, 50th, and 75th percentile values. To yield a cumulative measure of three PCDDs, we summed the ranks of the three POPs that belong to the PCDDs. The summary values were categorized by cutoff points of 25th, 50th, and 75th percentile values. We assigned and cumulated POP subclasses similarly for the three PCDFs, the four dioxin-like PCBs, the five non–dioxin-like PCBs, and the four OC pesticides.

The cross-sectional associations of categories of POPs with periodontal disease were analyzed using linear regression with continuous CAL%4 or PD%4 and logistic regression with dichotomous anyCAL4 or anyPD4. Potential confounders were age, sex, race/ethnicity, poverty income ratio, body mass index (BMI), cotinine concentration (nanograms per milliliter), history of cigarette smoking, and history of diabetes. We substituted median values computed from nonmissing data for participants with missing poverty income ratio, BMI, or cotinine concentration in 134 subjects; exclusion of these individuals did not change any conclusions. We also examined associations between POPs and subpopulations of WBC, specifically neutrophils and lymphocytes. To limit the possibility that inflammatory reaction due to various subclinical and clinical diseases affects these associations, we repeated the same analyses among subjects with C-reactive protein (CRP) < 3 mg/L (*n* = 744).

All statistical analyses were performed with SAS 9.1 (SAS Institute Inc., Cary, NC, USA) and SUDAAN 9.0 (Research Triangle Institute, Research Triangle Park, NC, USA). Estimates of the main results were calculated accounting for stratification and clustering (Korn and Graubard 1991), adjusting for age, race and ethnicity, and poverty income ratio instead of using sample weights. This adjustment has been regarded as a good compromise between efficiency and bias (Graubard and Korn 1999; Korn and Graubard 1991). Because results were very similar with SAS 9.1 and SUDAAN 9.0, we present the results based on SAS 9.1.

## Results

The sample of 1,243 participants included 45.5% men, 45.4% white, and 16.5% current smokers. Mean ± SD for age was 45.5 ± 17.6 years (range, 20–85). Table 1 summarizes the distribution of demographic or health behavior variables by the dichotomies anyCAL4 or anyPD4. Subjects with anyCAL4 tended to be older, male, smokers, and poor compared with those without anyCAL4. Subjects with anyPD4 showed a similar trend to that found with anyCAL4, except race; nonwhite race was common among subjects with anyPD4.

Table 2 shows the associations of five subclasses of POPs with demographic and health behavior factors. Age was the strongest correlate of serum concentrations of all five subclasses of POPs; correlation coefficients ranged from 0.45 to 0.88. Men tended to have lower concentrations of most POPs, except non–dioxin-like PCBs. White subjects had lower concentrations of OC pesticides but higher concentrations of PCBs and PCDFs. Mexican Americans had the highest serum concentrations of OC pesticides. Those with lower incomes had higher concentrations of OC pesticides but lower PCBs and PCDFs. Serum cotinine levels and current status of smoking were inversely associated with PCDDs while positively associated with non–dioxin-like PCBs. PCDDs were positively associated with BMI, whereas non–dioxin-like PCBs showed an inverse association with BMI.

Mean number ± SD of missing teeth was 4.4 ± 6.2, and 81.4% of study subjects had > 75% of teeth. The number of missing teeth was strongly associated with CAL; the correlation coefficient with CAL4% was 0.45, whereas that with PD4% was only 0.14. There were 298 prevalent cases of anyCAL4 (24.0%) and 205 prevalent cases of anyPD4 (16.5%). OC pesticides showed the strongest associations with anyCAL4 or anyPD4 (Table 3). After adjusting for age, sex, race/ethnicity, poverty income ratio, BMI, cigarette smoking, serum cotinine concentration, and diabetes, odds ratios (ORs) across quintiles of OC pesticides were 1.0, 1.3, 1.7, 2.4, and 2.7 (*p* for trend < 0.01) for anyCAL4 and 1.0, 1.7, 2.6, 3.4, and 3.7 (*p* for trend < 0.01) for anyPD4. Dioxin-like PCBs, non–dioxin-like PCBs, and PCDDs also showed significant positive associations with one or both definitions of periodontal disease. When we used continuous values of CAL%4 or PD%4, OC pesticides were most strongly associated under both definitions (Table 4). PCDDs and non–dioxin-like PCBs were significantly associated, but the relationships reached significance only with PD%4. Detailed associations of the four individual POPs belonging to OC pesticides with anyCAL4 or anyPD4 were presented in Table 5. Most specific POPs belonging to OC pesticides consistently showed associations with anyCAL4 or anyPD4.

**Figure 1:**
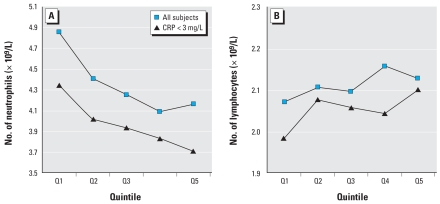
Neutrophil and lymphocyte counts by quintiles of OC pesticides adjusted for age, sex, race, poverty income ratio, cigarette smoking, serum cotinine concentration, and diabetes. *p*-Values for trend for neutrophils were < 0.01 both in all participants and in those with CRP < 3 mg/L.

We examined the association between OC pesticides and subtypes of differential WBC counts (Figure 1). Even though WBC and neutrophil counts were slightly higher among subjects with current periodontal disease, these counts generally had an inverse association with OC pesticides (*p* for trend < 0.01). This inverse association did not change after excluding subjects with CRP ≥ 3 mg/L and was even clearer after this exclusion in the last quintile of OC pesticides. Further adjustment for periodontal disease status did not change results (data not shown). On the other hand, there was a weak but not statistically significant positive trend of OC pesticides with lymphocyte counts. However, lymphocyte percentage of total white cells was significantly and positively associated with OC pesticides (data not shown).

## Discussion

In the present study we found that background exposure to POPs was positively associated with the prevalence of periodontal disease. This association was similarly observed using several definitions of periodontal disease, which could reflect various aspects of periodontal disease. Among five subclasses of POPs, OC pesticides were most strongly associated with periodontal disease. In fact, OC pesticides were the subgroup of POPs most strongly associated with type 2 diabetes, insulin resistance, and metabolic syndrome in our previous studies (Lee et al. 2006, 2007a, 2007b).

Because this is a cross-sectional study, temporality of events in any causal pathway that might link POPs exposure and periodontal disease cannot be established. However, it may be unlikely that periodontal disease increases serum concentrations of lipophilic xenobiotics that are mainly stored in adipose tissue, such as POPs. In addition, considering well-known immunomodulatory effects of endocrine disruptors such as POPs (Ahmed 2000) that were supported by the inverse association with neutrophil count, a relation in which POPs exposure leads to a predisposition to periodontal disease is biologically plausible.

In recent years, it has become apparent that the pathogenesis of periodontal disease is more complex than the simple presence of virulent microorganisms (Van Dyke and Serhan 2003). An individual′s susceptibility to periodontal breakdown depends on a number of identified and unidentified characteristics of the host, including the nonspecific and specific immune systems (Pattni et al. 2000). Immunodeficiency was suspected in patients exhibiting periodontal inflammation or destruction that appears disproportionate to the degree of local irritants (American Academy of Periodontology 2000). It is well known that patients who have innate impaired immune responses or who are taking immune suppressive medications are at a higher risk for periodontal disease (American Academy of Periodontology 2000).

Several lines of experimental and epidemiologic evidence have shown that chemicals such as POPs markedly influence the function of many of the cellular, subcellular, or molecular components of the immune system (Baccarelli et al. 2002; Banerjee 1999). An alteration of the normal immune function may have two types of consequences. The first is a reduction of immune activity, which can evolve into an immune deficit and increased susceptibility to infectious diseases and neoplasm. The second is an enhancement of the normal immune response, which can evolve into allergy and autoimmunity. In relation to autoimmunity, we reported positive associations between serum concentrations of some POPs, especially PCBs, and rheumatoid arthritis, an auto-immune disease (Lee et al. 2007c). The current study also suggests that exposure to background levels of POPs, especially OC pesticides, may increase susceptibility to bacterial infection.

In addition to periodontal disease and rheumatoid arthritis, background exposure to POPs was strongly and positively associated with many inflammatory chronic diseases such as type 2 diabetes, metabolic syndrome, and cardiovascular disease (Ha et al. 2007; Lee et al. 2006, 2007a, 2007b), and persons with these proinflammatory conditions generally have higher levels of WBC counts. However, the current study found that POPs, especially OC pesticides, were inversely associated with WBC counts, particularly neutrophil counts. This association was similarly observed among participants with CRP < 3 mg/L as well as among all participants. Although many infectious and inflammatory factors modulate changes in blood counts, the findings for POPs suggest that the basic hematopoietic process in bone marrow may be affected by POPs. There is a constant flow of neutrophils through the periodontal pocket, and they play a major role in the host response against invading periodontopathogenic microorganisms. As it is clear that deficiencies of the production rate or function of neutrophilic polymorphonuclear leukocytes can predispose to recurrent infections (Bogomolski-Yahalom and Matzner 1995), the subclinical decrease of neutrophils related to POPs exposure may predispose to bacterial infection in periodontal disease.

On the other hand, even though lymphocyte count was not associated with serum concentrations of OC pesticides, lymphocyte percentage was significantly and positively associated with them. It was impossible to examine this association in detail because of lack of information on subtypes of lymphocytes in the NHANES. In general, these findings about WBC counts could be interpreted to mean that the background exposure to OC pesticides may lead to a slight imbalance in the human immune system within the physiologic range, impairing the innate immune system but hyperactivating the acquired immune system. Long-term persistence of these features may lead to increased susceptibility to infection and autoimmune diseases among subjects with high exposure to OC pesticides.

Even though many epidemiologic studies have firmly established the association between periodontal disease and various systemic diseases (Demmer and Desvarieux 2006; Loe 1994), the nature and relevance of this association is still questionable (Paquette 2002). Specifically, it is an issue whether the infectious and inflammatory periodontal disease process contributes causally to various systemic diseases or these conditions are coincidentally associated with periodontal disease (Paquette 2002). Although inflammatory reactions in the periodontal region have been associated with the risk of various systemic diseases, our studies on POPs also suggest that the simultaneous exposure to environmental xenobiotics such as POPs, which consist of several hundred chemicals with various harmful effects, may contribute to the pathogenesis of these clinical outcomes, leading to the epidemiologic associations between periodontal disease and various systemic diseases.

This study has several limitations. First, the cross-sectional study design in NHANES does not allow inferences regarding the causality between POPs and periodontal disease, despite biological plausibility of our findings. Next, misclassification bias is possible for subjects whose POPs would have been detectable with a higher sample volume. However, such misclassification would be nondifferential if sample volume was unrelated to periodontal disease. Finally, there can be residual confounding due to unknown lifestyle factors, which can affect both body burden of POPs and periodontal disease. However, the findings presented do adjust for several lifestyle factors.

Taken together, serum concentrations of POPs, especially OC pesticides, were positively associated with periodontal disease. Immunomodulation due to exposure to OC pesticides may be a contributory mechanism.

## Figures and Tables

**Table 1:** Comparison of demographic and health behavior factors between subjects with or without CAL ≥ 4 mm in any site and with or without PD ≥ 4 mm in any site.

	CAL			PD		
Demographic/health factor	Absence	Presence	*p-*Value	Absence	Presence	*p-*Value
No. of subjects	945	298		1,038	205	
Age (years)	41.6 ± 16.6	57.8 ± 14.9	< 0.01	45.0 ± 17.8	47.9 ± 16.5	0.03
Poverty income ratio	2.7 ± 1.6	2.3 ± 1.5	< 0.01	2.7 ± 1.6	2.2 ± 1.4	< 0.01
BMI (kg/m^2^)	28.3 ± 6.2	28.1 ± 5.7	0.60	28.2 ± 6.1	28.4 ± 6.0	0.76
Serum cotinine (ng/mL)	38.1 ± 94.7	61.0 ± 114.8	< 0.01	39.6 ± 93.8	64.1 ± 126.5	< 0.01
Proportion (%)						
Male	40.7	60.4	< 0.01	43.0	58.1	< 0.01
White	45.2	46.0	0.81	49.1	26.3	< 0.01
Smoker	14.5	22.8	< 0.01	15.3	22.4	0.01

Values are mean ± SD except where indicated.

**Table 2:** Age-adjusted Spearman correlation coefficients^a^ between each of five categories of lipid-adjusted POPs (three PCDDs, three PCDFs, four dioxin-like PCBs, five non–dioxin-like PCBs, and four OC pesticides) and demographic and health behavior factors.

Demographic/health factor	PCDDs	PCDFs	Dioxin-like PCBs	Non–dioxin-like PCBs	OC pesticides
Age	0.55**	0.44**	0.70**	0.72**	0.73**
Sex (male = 1; female = 0 )	−0.16**	0.01	−0.10**	0.08**	−0.07*
Race (white = 1; others = 0)	−0.04	0.08**	0.09**	0.11**	−0.33**
Poverty income ratio	−0.01	0.08**	0.12**	0.16**	−0.17**
Serum cotinine level	−0.13**	0.02	−0.05	0.13**	−0.02
Cigarette smoking (current = 1; others = 0)	−0.14**	0.01	−0.05	0.10**	0.02
BMI	0.13**	0.03	0.03	−0.13**	0.03

a Before calculating correlation coefficients, detectable values of each POP were individually ranked, and the ranks were summed within subclass to arrive at the subclass value. All not detectable values were ranked as zero.

* *p* < 0.05.

** *p* < 0.01.

**Table 3:** Adjusted^a^ ORs [95% confidence intervals (CIs)] of prevalence of CAL or PD by quintiles of PCDDs, PCDFs, dioxin-like PCBs, non–dioxin-like PCBs, and OC pesticides.

	Quintiles of POPs^b^					
Indicator/compound	< 20th	20th to < 40th	40th to < 60th	60th to < 80th	≥ 80th	*p-*Trend
CAL ≥ 4 mm (presence vs. absence in any site)						
PCDDs						
Cases/no.	35/248	42/250	52/248	69/249	100/248	
Adjusted OR (95% CI)	Referent	1.0 (0.6–1.8)	1.1 (0.7–1.9)	1.3 (0.8–2.3)	1.7 (0.9–3.0)	0.04
PCDFs						
Cases/no.	36/248	45/249	50/249	75/249	92/248	
Adjusted OR (95% CI)	Referent	1.1 (0.6–1.8)	1.0 (0.6–1.7)	1.3 (0.8–2.2)	1.3 (0.8–2.2)	0.18
Dioxin-like PCBs						
Cases/no.	16/248	46/250	54/248	80/249	102/248	
Adjusted OR (95% CI)	Referent	1.9 (1.0–3.6)	2.0 (1.0–3.8)	2.3 (1.2–4.4)	2.3 (1.1–4.6)	0.06
Non–dioxin like PCBs						
Cases/no.	25/248	25/249	50/249	86/249	112/248	
Adjusted OR (95% CI)	Referent	0.9 (0.5–1.8)	1.3 (0.7–2.2)	1.7 (0.9–3.1)	1.8 (0.9–3.3)	0.03
OC pesticides						
Cases/no.	17/248	30/250	53/248	86/249	112/248	
Adjusted OR (95% CI)	Referent	1.3 (0.7–2.4)	1.7 (0.9–3.2)	2.4 (1.2–4.6)	2.7 (1.3–5.5)	< 0.01
PD ≥ 4 mm (presence vs. absence in any site)						
PCDDs						
Cases/no.	35/248	40/250	39/248	43/249	48/248	
Adjusted OR (95% CI)	Referent	1.3 (0.8–2.2)	1.3 (0.8–2.2)	1.4 (0.8–2.5)	1.7 (1.0–3.1)	0.10
PCDFs						
Cases/no.	31/248	43/249	41/249	51/249	39/248	
Adjusted OR (95% CI)	Referent	1.4 (0.9–2.4)	1.3 (0.8–2.2)	1.6 (1.0–2.7)	1.1 (0.6–2.0)	0.49
Dioxin-like PCBs						
Cases/no.	27/248	44/250	44/248	40/249	50/248	
Adjusted OR (95% CI)	Referent	1.5 (0.9–2.6)	1.9 (1.1–3.3)	1.6 (0.9–3.1)	2.4 (1.2–4.7)	0.03
Non–dioxin like PCBs						
Cases/no.	31/248	42/249	32/249	47/249	53/248	
Adjusted OR (95% CI)	Referent	1.4 (0.9–2.4)	1.0 (0.6–1.8)	1.6 (0.9–2.9)	1.8 (1.0–3.5)	0.08
OC pesticides						
Cases/no.	21/248	33/250	46/248	53/249	52/248	
Adjusted OR (95% CI)	Referent	1.7 (0.9–3.0)	2.6 (1.4–4.8)	3.4 (1.8–6.5)	3.7 (1.8–7.6)	< 0.01

a Adjusted for age, sex, race, poverty income ratio, serum cotinine levels, cigarette smoking, and diabetes.

b Detectable values of each POP were individually ranked, and the ranks were summed within subclass to arrive at the subclass value. All not detectable values were ranked as zero. The summary values were categorized by quintiles of the sum of ranks.

**Table 4:** Adjusted^a^ means (SEs) of CAL percentage of sites ≥ 4 mm or PD percentage of sites ≥ 4 mm by quartiles of PCDDs, PCDFs, dioxin-like PCBs, non–dioxin-like PCBs, and OC pesticides.

	Quintiles of POPs^b^					
	< 20th	20th to < 40th	40th to < 60th	60th to < 80th	≥ 80th	*p*-Trend
CAL % of sites ≥ 4 mm						
PCDDs	7.0 (1.1)	5.9 (1.1)	7.4 (1.1)	7.1 (1.1)	8.5 (1.2)	0.30
PCDFs	6.8 (1.1)	6.7 (1.1)	5.9 (1.1)	8.8 (1.1)	7.8 (1.1)	0.27
Dioxin-like PCBs	6.0 (1.2)	6.5 (1.1)	6.5 (1.1)	8.6 (1.1)	8.4 (1.3)	0.16
Non–dioxin-like PCBs	6.4 (1.2)	5.3 (1.2)	6.0 (1.1)	8.3 (1.1)	10.0 (1.3)	0.06
OC pesticides	6.0 (1.3)	4.7 (1.1)	5.1 (1.1)	9.0 (1.1)	11.1 (1.3)	< 0.01
PD % of sites ≥ 4 mm						
PCDDs	2.3 (0.8)	2.7 (0.8)	4.1 (0.8)	3.8 (0.8)	5.7 (0.8)	< 0.01
PCDFs	2.8 (0.8)	3.7 (0.8)	2.9 (0.8)	5.8 (0.8)	3.5 (0.8)	0.16
Dioxin-like PCBs	2.2 (0.9)	3.0 (0.8)	5.0 (0.8)	3.7 (0.8)	4.7 (0.9)	0.07
Non–dioxin-like PCBs	1.9 (0.9)	3.7 (0.8)	2.6 (0.8)	4.5 (0.8)	6.0 (0.9)	< 0.01
OC pesticides	1.1 (0.9)	2.7 (0.8)	4.1 (0.8)	4.9 (0.8)	5.9 (0.9)	< 0.01

a Adjusted for age, sex, race, poverty income ratio, serum cotinine levels, cigarette smoking, and diabetes.

b Detectable values of each POP were individually ranked and the rank orders of the individual POPs in each subclass were summed to arrive at the subclass value. All not detectable values were ranked as zero. The summary values were categorized by quintiles of the sum of ranks.

**Table 5:** Adjusted^a^ ORs (95% CIs) of prevalence of any CAL ≥ 4 mm or PD ≥ 4 mm by quintiles (Q) of specific POPs belonging to OC pesticides.

		Detectable					
	Nondetectable	Q1	Q2	Q3	Q4	Q5	*p*-Trend
CAL ≥4 mm (presence vs. absence in any site)							
Oxychlordane	Referent	1.4 (0.8–2.3)	1.1 (0.6–2.0)	1.9 (1.1–3.5)	1.7 (0.9–3.3)	2.3 (1.2–4.5)	0.01
*trans*-Nonachlor	Referent	3.3 (1.3–8.3)	2.6 (1.0–6.6)	3.9 (1.6–9.9)	4.2 (1.6–10.7)	5.0 (1.9–13.3)	< 0.01
*p,p*′-DDE^b^		Referent	1.4 (0.8–2.3)	1.0 (0.6–1.7)	1.6 (0.9–2.8)	1.7 (0.9–3.0)	0.07
β-HCH	Referent	1.3 (0.7–2.3)	2.2 (1.3–3.7)	1.5 (0.8–2.7)	2.2 (1.2–4.0)	2.0 (1.0–3.8)	0.02
PD ≥ 4 mm (presence vs. absence in any site)							
Oxychlordane	Referent	1.3 (0.7–2.3)	1.6 (0.9–2.8)	1.6 (0.9–3.0)	1.5 (0.8–2.9)	2.6 (1.3–5.2)	0.02
*trans*-Nonachlor	Referent	1.9 (1.0–3.6)	1.9 (1.0–3.8)	2.1 (1.1–4.3)	2.1 (1.0–4.4)	2.4 (1.1–5.2)	0.05
*p,p*′-DDE^b^		Referent	1.2 (0.7–2.0)	1.6 (0.9–2.8)	2.2 (1.2–3.8)	2.2 (1.2–4.0)	< 0.01
β-HCH	Referent	1.6 (0.9–2.9)	3.3 (2.0–5.7)	2.0 (1.0–3.7)	2.4 (1.2–4.5)	4.1 (2.1–7.9)	< 0.01

Abbreviations: *p,p*′-DDE, *p,p*′-dichlorodiphenyltrichloroethane; β-HCH, beta-hexachlorocyclohexane.

a Adjusted for age, sex, race, poverty income ratio, cigarette smoking, serum cotinine, and diabetes.

b *p,p*′-DDE were detected in all subjects.
